# Editorial: Microbial biomarkers predicting BCG therapy response in non-muscle invasive bladder cancer

**DOI:** 10.3389/fonc.2026.1831100

**Published:** 2026-04-17

**Authors:** Guglielmo Mantica, Francesco Esperto, Rafaela Malinaric

**Affiliations:** 1 Department of Surgical and Diagnostic Integrated Sciences (DISC), University of Genova, Genova, Italy; 2 Department of Urology, Campus Bio-Medico University, Rome, Italy; 3 IRCCS Azienda Ospedaliera Metropolitana, Genova, Italy

**Keywords:** BCG, bladder cancer, microbial, NMIBC, TURBT

Non-muscle invasive bladder cancer (NMIBC) represents about 70–75% of newly diagnosed bladder cancers (BC) and is typically managed with transurethral resection of bladder tumor (TURBT) followed by intravesical therapy in patients at intermediate or high risk of recurrence and progression. Intravesical Bacillus Calmette–Guérin (BCG) remains the most effective adjuvant immunotherapy for NMIBC and has been widely used for decades (1). Despite its clinical success, however, a substantial proportion of patients fail to respond adequately to treatment or experience recurrence after therapy. To identify some reliable biomarkers capable of predicting response to BCG remains one of the most pressing challenges in the management of NMIBC (2).

In recent years, there have been an increasing attention toward the role of host immunity and microbial ecosystems in shaping responses to cancer and BC immunotherapy (3). The human body hosts complex microbial communities that influence both innate and adaptive immune responses. Advances in sequencing technologies have revealed that the urinary tract is not sterile, as previously believed, but instead harbors a diverse microbiome that may interact with the tumor microenvironment and modulate therapeutic responses.

In this light, the Research Topic “Microbial Biomarkers Predicting BCG Therapy Response in Non-Muscle Invasive Bladder Cancer” aims to explore novel biomarkers and immunological mechanisms associated with BCG responsiveness. The articles included in this collection highlight emerging insights into host immune responses, systemic inflammatory markers, and potential biological indicators that may help predict treatment outcomes.

One important aspect addressed in this collection is the role of tumor-specific immune responses in NMIBC. In their study, Brochier et al. investigated spontaneous anti-neoepitope CD8^+^ T-cell responses in patients with bladder cancer. By combining functional assays with transcriptomic analyses of tumor-infiltrating lymphocytes, the authors demonstrated that a subset of patients harbors detectable neoantigen-specific T-cell responses in blood and tumor tissues. Their findings highlight the immunogenic nature of bladder cancer and suggest that pre-existing antitumor immune responses may influence the effectiveness of BCG therapy.

Another study examined the dynamics of tuberculin purified protein derivative (PPD)-specific T-cell responses during BCG therapy. Duan et al. evaluated peripheral immune responses in NMIBC patients undergoing intravesical BCG instillation, demonstrating that treatment induces measurable changes in antigen-specific T-cell reactivity over time. These findings support the concept that systemic immune monitoring may provide valuable information about the biological activity of BCG therapy and could potentially serve as a biomarker of therapeutic response or immune activation during treatment.

In addition to tumor-specific immune responses, systemic inflammatory markers have also emerged as potential predictors of clinical outcomes in NMIBC. Li et al. investigated the lymphocyte-to-monocyte ratio (LMR) and demonstrated that the ratio measured during BCG induction may provide stronger prognostic value than baseline measurements alone. Dynamic changes in inflammatory biomarkers during treatment may therefore offer insight into the host immune response and its relationship with therapeutic efficacy.

Similarly, previous analyses have shown that elevated systemic inflammatory markers, such as the neutrophil-to-lymphocyte ratio (NLR), are associated with poorer outcomes in NMIBC patients undergoing intravesical BCG therapy. These findings further support the hypothesis that systemic immune status plays a critical role in determining treatment response and disease progression. Collectively, such markers represent attractive candidates for easily accessible and cost-effective predictive tools in routine clinical practice.

Taken together, the studies included in this Research Topic highlight the complexity of the immune landscape in NMIBC and emphasize the multifaceted nature of BCG therapy response. While BCG has long been recognized as a potent immunotherapeutic agent, the mechanisms underlying its clinical efficacy remain incompletely understood. The interaction between tumor cells, host immune responses, and microbial communities within the urinary tract may play a crucial role in shaping treatment outcomes. The identification of reliable predictive biomarkers is particularly important given the substantial heterogeneity observed in clinical responses to BCG. Early identification of patients unlikely to benefit from BCG therapy could facilitate more timely adoption of alternative treatments, including radical cystectomy or emerging bladder-sparing strategies such as immune checkpoint inhibitors and novel intravesical therapies (4). In addition, future investigations should place greater emphasis on the early identification of BC histological rare variants, which may underlie heterogeneous immune responses to BCG and represent overlooked determinants of treatment resistance in specific patient subsets (5).

Future research will likely benefit from integrative approaches combining microbiome profiling, immune monitoring, and molecular characterization of tumors. In addition, prospective studies incorporating longitudinal sampling of urine, blood, and tumor tissue will be essential to validate candidate biomarkers and clarify their clinical utility. In conclusion, the contributions presented in this Research Topic provide valuable insights into the immunological and biological factors associated with BCG therapy response in NMIBC. By exploring novel immune and microbial biomarkers, these studies contribute to a growing body of evidence supporting a more personalized approach to bladder cancer management. Continued investigation into the complex interplay between host immunity, microbial ecosystems, and tumor biology may ultimately lead to improved predictive tools and more effective therapeutic strategies for patients with NMIBC.

## References

[1] Lu LL Chen HZ Chen JG . Impact of age, TNM stage, and hospitalization on bladder cancer survival: evidence from a hospital-based cohort in eastern China. Res Rep Urol. (2025) 17:481–93. doi: 10.2147/RRU.S568396 PMC12691630 41384284

[2] Malinaric R Mantica G Lo Monaco L Mariano F Leonardi R Simonato A . The role of novel bladder cancer diagnostic and surveillance biomarkers-what should a urologist really know? Int J Environ Res Public Health. (2022) 19:9648. doi: 10.3390/ijerph19159648 35955004 PMC9368399

[3] Zhu Q Zhang G Cao M Huang H He D Zang Z . Microbiota-shaped neutrophil senescence regulates sexual dimorphism in bladder cancer. Nat Immunol. (2025) 26:722–36. doi: 10.1038/s41590-025-02126-6 40217111

[4] Feng R Zhang J Tao Y Hou J Tai W . Preoperative inflammation-based immune prognostic nomogram in bladder cancer: a multicenter study. Front Oncol. (2026) 15:1644747. doi: 10.3389/fonc.2025.1644747 41613549 PMC12847027

[5] Mantica G Simonato A Du Plessis DE Maffezzini M De Rose AF van der Merwe A . The pathologist’s role in the detection of rare variants of bladder cancer and analysis of the impact on incidence and type detection. Minerva Urol Nefrol. (2018) 70:594–7. doi: 10.23736/S0393-2249.18.03175-2 30203936

